# Supplementary material for: Global Transcriptional Response of Three Highly Acid-Tolerant Field Strains of Listeria monocytogenes to HCl Stress
Source: Microorganisms. 2019 Oct 16;7(10):455. doi: 10.3390/microorganisms7100455 (PMC6843411; doi:10.3390/microorganisms7100455)
Supplement: Supplementary file 1 [file microorganisms-07-00455-s001.zip › supplementary/Supplementary figures.docx]

| 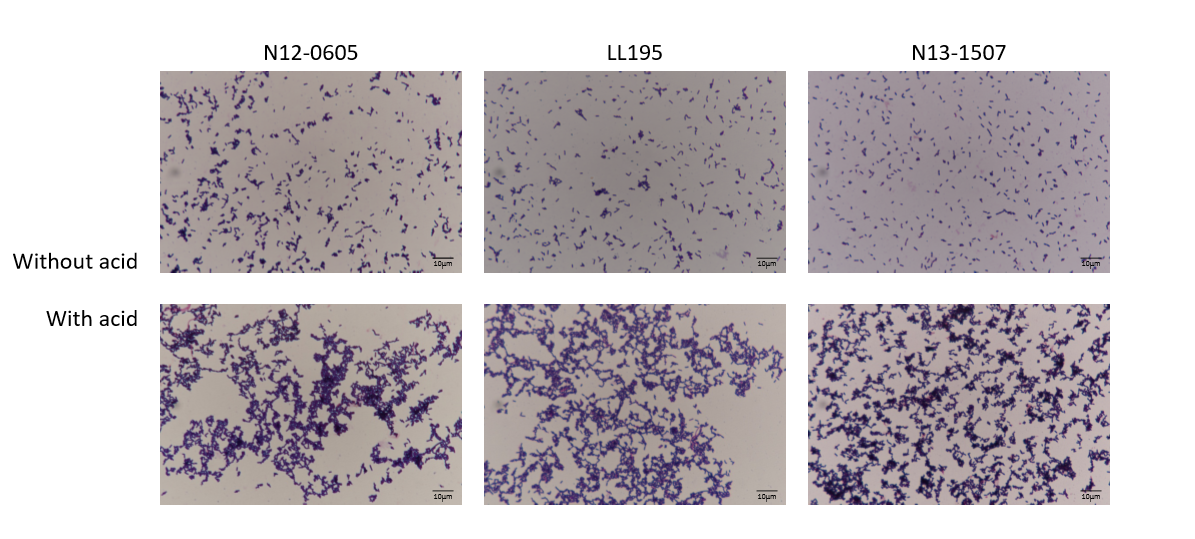 |
| --- |

**Supplementary Figure 1:** Gram stains of the *L. monocytogenes* strains used in this study, before and after acid stress with HCl at pH 3 for 1h. Cells were fixed with 1% paraformaldehyde and stained with the standard protocol for Gram staining.

| 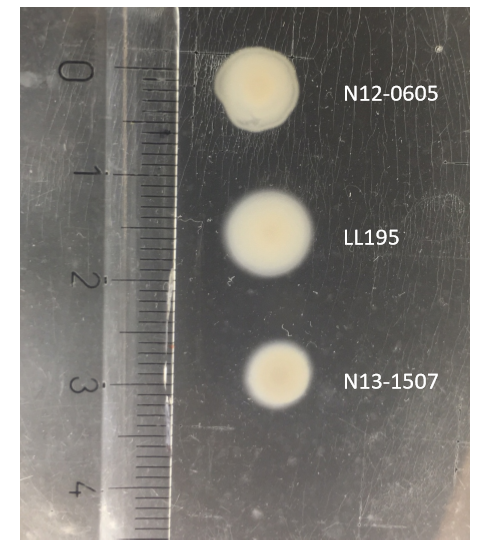 |
| --- |

**Supplementary Figure 2:** A fine pipette tip was dipped into overnight cultures grown at 37°C and used to inoculate a small focus into the soft agar.
